# Recombinant protein HR212 targeting heptad repeat 2 domain in spike protein S2 subunit elicits broad‐spectrum neutralizing antibodies against SARS‐CoV‐2 and its variants

**DOI:** 10.1002/mco2.70088

**Published:** 2025-02-09

**Authors:** Ying Lu, An‐Qi Li, Fan Shen, Wen‐Qiang He, Shu‐Heng Yu, Yan‐Bo Zhao, Xiao‐Li Feng, Ming‐Hua Li, Songying Ouyang, Yong‐Tang Zheng, Wei Pang

**Affiliations:** ^1^ State Key Laboratory of Genetic Evolution & Animal Models, Key Laboratory of Bioactive Peptides of Yunnan Province, KIZ‐CUHK Joint Laboratory of Bioresources and Molecular Research in Common Diseases, Kunming Institute of Zoology, Chinese Academy of Sciences Kunming Yunnan China; ^2^ University of Chinese Academy of Sciences Beijing China; ^3^ Department of Biochemistry and Molecular Biology Faculty of Basic Medical Science Kunming Medical University Kunming Yunnan China; ^4^ The Key Laboratory of Innate Immune Biology of Fujian Province, Provincial University Key Laboratory of Cellular Stress Response and Metabolic Regulation, Biomedical Research Center of South China, Key Laboratory of OptoElectronic Science and Technology for Medicine of Ministry of Education, College of Life Sciences, Fujian Normal University Fuzhou China; ^5^ Kunming National High‐level Biosafety Research Center for Non‐human Primates, Center for Biosafety Mega‐Science, Kunming Institute of Zoology, Chinese Academy of Sciences Kunming Yunnan China; ^6^ EterniVax Biomedical Inc Shanghai China; ^7^ Department of Pathogen Biology and Immunology Faculty of Basic Medical Science Yunnan Provincial Key Laboratory of Public Health and Biosafety, Kunming Medical University Kunming China

**Keywords:** broadly neutralizing antibodies, HR1 domain, HR2 domain, S2 subunit, SARS‐CoV‐2 variants

## Abstract

SARS‐CoV‐2 variants are under continuous emergence carry numerous mutations within S1 subunit in spike protein and have escaped neutralization through many currently used vaccines and antibodies. The development of next‐generation vaccines is a continuing and long‐term need. In our prior research, the recombinant protein vaccine HR121 targeting the heptad repeat (HR) 1 domain of S2 subunit was constructed, which could evoke highly broad‐spectrum neutralizing antibodies in vivo and confer efficient protective effect on several SARS‐CoV‐2 variants within multiple animal models. Compared with HR1, HR2 domain shows a more conservative degree within SARS‐CoV‐2 and related coronaviruses. Here, we designed a recombinant protein HR212 consisting of HR2–linker1–HR1–linker2–HR2. HR212 showed a high affinity with HR1 and was functionally analogous to HR2 within fusion intermediate in S2 subunit. Immunizing rabbits using HR212‐mediated high nAbs for 28 pseudotyped SARS‐CoV‐2 variants, like currently circulating variants, such as BA.2.86 and JN.1. Transfer of rabbit anti‐HR212 sera or immunization with HR212 offered efficient protective effect on SARS‐CoV‐2 ancestral strain and Omicron BA.2 variant infections of Syrian golden hamsters. According to our results, HR2 domain of S2 subunit is the novel target that can be used to develop broad‐spectrum vaccines to resist SARS‐CoV‐2 variants.

## Graphical abstract

1. HR212 assembles to be a trimer with three HR2s exposed.

2. The nAbs induced by HR212 showed inhibition of HR1 binding with HR212 and the nAbs induced by HR212 would block the S2 conformational rearrangement from “fusion intermediate” to “postfusion” structure (6‐HB), thus inhibiting membrane fusion between viral and cellular membranes.

3. Immunization with HR212 evoked highly cross‐nAbs against SARS‐CoV‐2 and its variants.

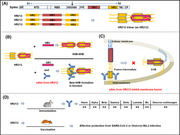

## 1 INTRODUCTION

The SARS‐CoV‐2 pandemic and its subsequently emerging variants have posed major challenges to pandemic control.^1^, ^2^, ^3^, ^4^

During infection, SARS‐CoV‐2 first enters host cells, which serves as the main target for prophylactic and therapeutic designs. Like other coronaviruses, spike (S) protein in SARS‐CoV‐2 mediates viral entry in cells by combining with host receptor angiotensin‐converting enzyme 2 (ACE2) via the S1 subunit, then promoting membrane fusion via its S2 subunit.^5^ Currently, S1 subunit, especially the N‐terminal domain and receptor binding domain (RBD), triggers the main neutralizing antibodies (nAbs), which is the prime antigen in vaccine designs.^4^ Unfortunately, under the selective pressure from the host, many mutations in SARS‐CoV‐2 variants are within S1 subunit, particularly in the RBD domain, which causes significant immune escapes of the variants from neutralization by most currently approved vaccines, resulting in continuous breakthrough infection.^6^ Thus, strategies for vaccine designs targeting the more conserved region are urgently needed.

S2 subunit is embedded in S1 subunit within the conformation before fusion. S2 contains two helix domains (HR), HR1 and HR2, which display high conservation degree within most coronaviruses. During fusion stage, HR1 and HR2 within S2 subunit are under immediate exposure to form the “fusion intermediate” conformation. Subsequently, the three HR2s roll back anti‐parallelly and fold within surface grooves in HR1 trimeric inner core to form the irreversible six‐helical bundle (6‐HB) and promoting viral‐cellular membrane fusion.^7^, ^8^ During this process, some HR2‐derived peptides, like EK1 and EK1C, can potently combine with HR1 trimer and block 6‐HB generation, thus exerting antiviral effect on multiple SARS‐CoV‐2 variants or more coronaviruses.^9^, ^10^, ^11^ Therefore, the conserved HR1 and HR2 domains within the S2 subunit are potent targets for pan‐coronavirus inhibitor.

On the other side, in our prior work, the recombinant protein vaccine HR121 was constructed, and it can bind to HR2. In several rodent and rhesus macaque models, this protein can evoke highly broad‐spectrum nAbs targeting HR1 domain within S2 subunit and provide protections against SARS‐CoV‐2 as well as the corresponding variant infections. Therefore, the conserved HR domains can also be developed as promising targets for pan‐SARS‐CoV‐2 vaccine designs.^12^

Relative to HR1 domain, HR2 domain is more conserved in most coronaviruses.^10^ For example, the HR2 shows 100% identity in amino acid sequence among SARS‐CoV, SARS‐CoV‐2, WIV1, and HKU3, while HR1 in SARS‐CoV‐2 shows 90, 90, and 98.9% amino acid identity to the counterparts of SARS‐CoV, WIV1, and HKU3,^13^ respectively. This suggests that the HR2 domain may be a more suitable target for pan‐coronavirus inhibitor or vaccine designs. Although some HR1 derived fusion inhibitors exhibited HR2‐binding activities and showed broad‐spectrum neutralization for SARS‐CoV‐2 together with the variants, there have been no reported nAbs or vaccines targeting this domain.^14^ In the present study, analogous to HR121, we designed another recombinant protein HR212. Immunizing rabbits with HR212 induced broadly nAbs targeting HR2 domain within S2 subunit. Transferring rabbit anti‐HR212 sera or immunization with HR212 effectively prevented Syrian golden hamsters against SARS‐CoV‐2 ancestral strain or Omicron BA.2 variant infections, the pathological lesions and pulmonary viral loads decreased within immunized hamsters. These findings indicate that the HR2 within S2 subunit in SARS‐CoV‐2 is the new target for developing broad‐spectrum vaccines to resist SARS‐CoV‐2 together with the emerging variants.

## 2 RESULTS

### 2.1 Recombinant protein HR212 preparation and characteristics

**FIGURE 1:**
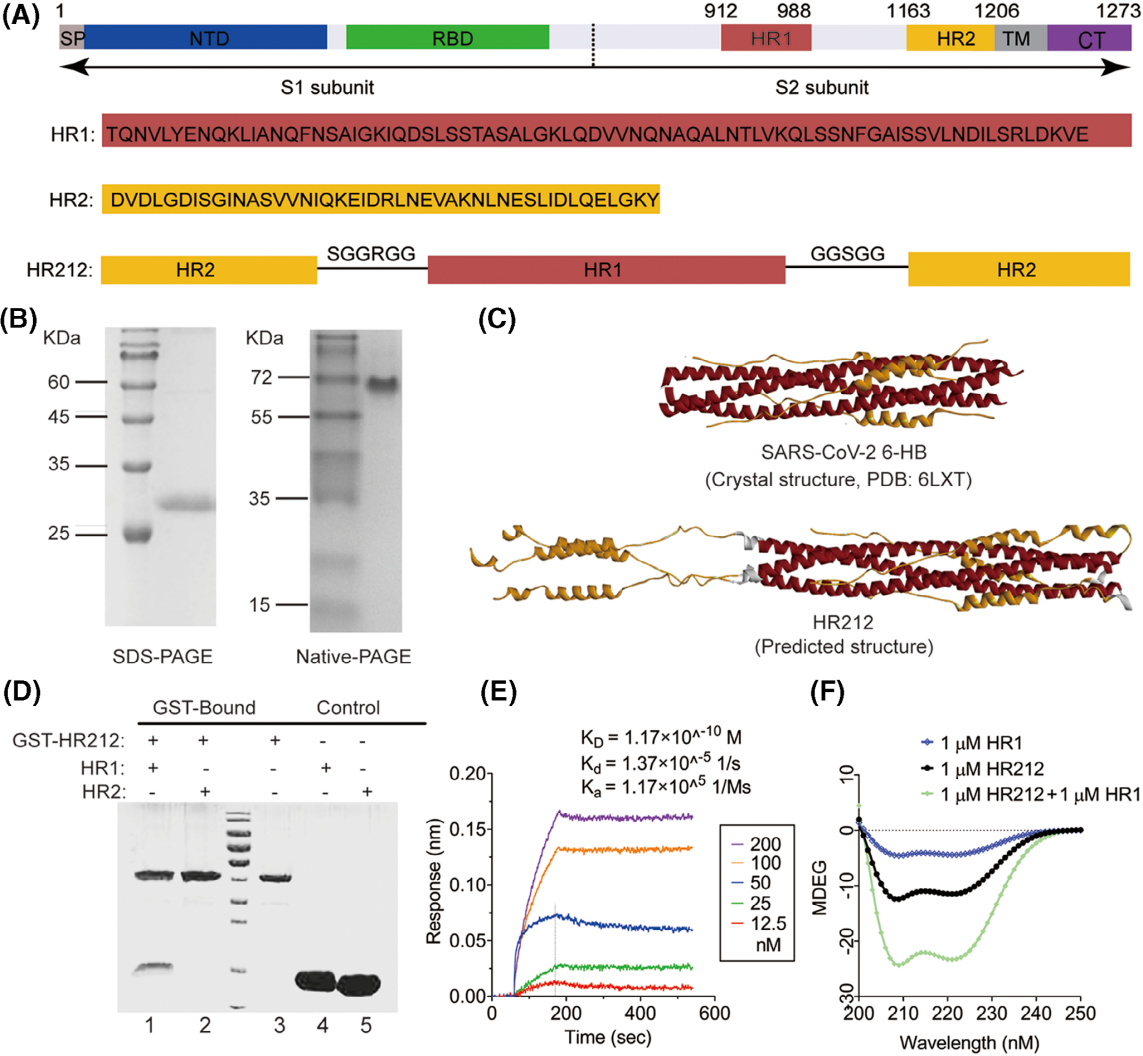
HR212 could bind with HR1 to form a more stable α‐helix. (A) Amino acid sequence of HR212. HR2 and HR1 were derived from spike protein of SARS‐CoV‐2 Wuhan hu‐1 strain (GenBank No. NC_045512.2). HR212 monomer is composed of HR2−linker 1−HR1−linker 2−HR2. (B) Identification of purified HR212 protein by PAGE electrophoresis and gel filtration chromatography (Superdex 200 Increase 10/300 GL column). (C) The crystal structure of SARS‐CoV‐2 6‐HB (PDB entry 6LXT) and predicted structure of HR212 formed by the HR1 domain (red), linker (white) and HR2 domain (yellow). (D) GST pull‐down assay showed that HR212 selectively bound to HR1. Lane 1: beads‐GST‐HR212 + HR1; Lane 2: beads‐GST‐HR212 + HR2; Lane 3: beads‐GST‐HR212; Lane 4: beads + HR1; Lane 5: beads + HR2. (E) Surface plasmon resonance analysis of the binding affinity between HR1 and HR212. (F) CD spectrum detected that HR212 could bind with HR1 to form a more stable α‐helix. MDEG: millidegree. 6HB: six‐helical bundle.

Similar to the construction of previously reported recombinant protein vaccine HR121,^12^ here, using the HR1 and HR2 full sequences, we design a new recombinant protein that mimics HR2 in S2 subunit. This protein includes HR2–linker 1–HR1–linker 2–HR2 and is termed as HR212 (Figure 1A). HR212 showed abundant expression within *E. coli* strain BL21 (DE3), which was under purification through the one‐step Ni chromatography column, yielding a high purity. The obtained protein was determined to be approximately 28 kDa in SDS‐PAGE. However, it was shown an oligomeric state in the elution of and was detected to be a trimer in Naive‐PAGE (Figure 1B). To further understand its structure, we constructed a protein model of HR212 using Alpha Fold analysis and compared with 6‐HB structure for SARS‐CoV‐2 (6LXT). As expected, HR212 self‐assembled to form a trimer, wherein three HR12s formed a 6‐HB structure, which is same as the 6LXT, while the additional three free HR2s parallelly linked at the N‐terminal of the HR1 trimer in 6‐HB (Figure 1C). As shown in the GST‐pulldown assay, HR212 can interact only with HR1, but not with HR2 (Figure 1D). Surface plasmon resonance experiment demonstrated that the dissociation constant (*K*_D_) between HR212 and HR1 was 1.2 × 10^−10^  M, with the association rate constant (ka) and dissociation rate constant (kd) being 1.2 × 10^5^/Ms and 1.4×10^−5^/s. The binding affinity between HR212 and HR1 was comparable to that between HR2 and HR1,^7^ suggesting that HR212 is functionally analogous to HR2, can bind to HR1 with high affinity (Figure 1E). Circular dichroism spectra revealed that HR212 had a notable α‐helix feature, and it interacted with HR1 for forming the stable α‐helix structure (Figure 1F). Therefore, analogous to the HR121 vaccine, wherein the main nAbs were induced by the exposed HR1 domain, but not by the 6‐HB (HR12) structure,^12^ we speculate that part of the antibodies induced by HR212 protein would target the three free HR2s in the HR212 trimer, and inhibit HR1 and HR2 binding in the process of membrane fusion, thereby blocking virus from entering host cells.

### 2.2 HR212 induced high levels of nAbs for SARS‐CoV‐2 as well as the relevant variants within rabbits

**FIGURE 2:**
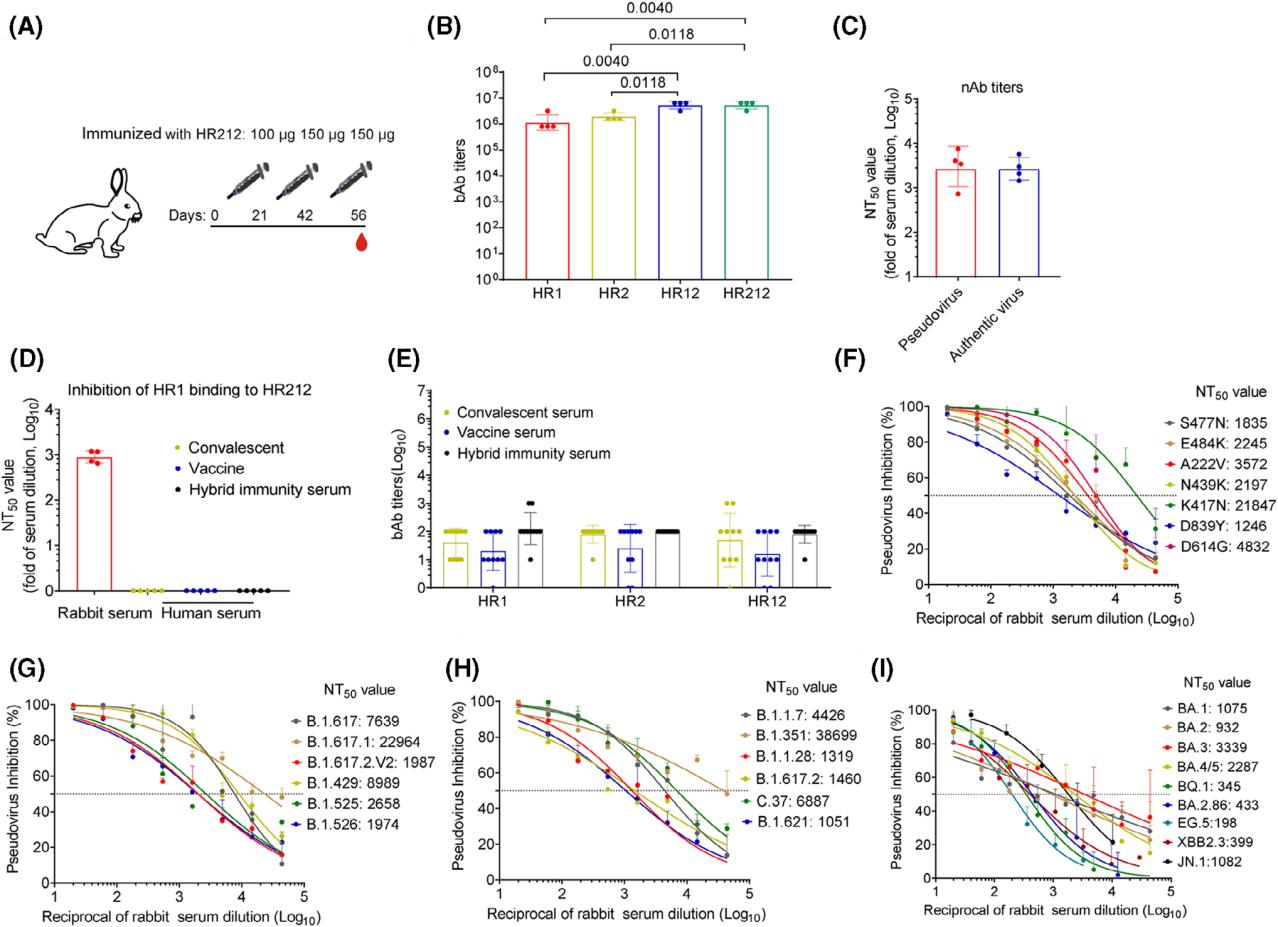
HR212 induced a high effective neutralizing antibody against SARS‐CoV‐2 variants in rabbits. (A) Schematic diagram of rabbit immunized with HR212. (B) End‐point titers of HR1, HR2, HR12, and HR212 protein binding antibodies (bAbs) in HR212 immunized rabbits after the third immunization. (C) The titers of neutralizing antibodies (nAbs) against SARS‐CoV‐2 ancestral strain pseudovirus and authentic viruses in rabbit sera immunized with HR212 (*n* = 4). (D) When serum‐HR1‐HRP mixture were added to the coated HR212, rabbit anti‐HR212 sera (*n* = 4) could inhibit the binding between HR212 and HR1, while the sera from convalescent (*n* = 5), vaccinated (*n* = 5), and hybrid immunity (*n* = 5) humans could not inhibit the HR212 and HR1 binding in the competitive ELISA. (E) End‐point titers of bAbs against HR1, HR2, and HR12 in convalescent (*n* = 10), vaccinated (*n *= 10), and hybrid immunity (*n* = 10) humans, the serum samples in Figure 2D were randomly selected from the samples in Figure 2E. (F–I) Inhibitory effect of rabbit anti‐HR212 sera against multiple SARS‐CoV‐2 variants, three independently repeated neutralization experiments were carried out. Data are presented as geometric mean ± geometric SD and differences between each group are determined by one‐way ANOVA.

For validating this hypothesis, we subcutaneously injected four rabbits with HR212 in Freund's adjuvant thrice every 3 weeks (Figure 2A). Similar to the antibody production in rabbits immunized with HR121, HR212 induced high titers of antibodies to itself (geometric mean titer [GMT]: 5.4 × 10^6^), followed by 6‐HB scaffold (HR12) antibodies (GMT: 5.4 × 10^6^), and HR1 and HR2 antibody titers were lower than them (GMTs: 1.1 × 10^6^ and 2.0 × 10^6^) (Figure 2B). To determine the antiviral activity of these antibodies, we added serial dilutions of rabbit anti‐HR212 sera to 293T‐ACE2 cells and evaluated their inhibition on ancestral SARS‐CoV‐2 pseudoviruses that entered the cells. The anti‐HR212 sera efficiently neutralized SARS‐CoV‐2 pseudoviruses, and the geometric mean 50% neutralization titer (GMT) was 2.9 × 10^3^ (Figure 2C). Rabbit anti‐HR212 sera were evaluated for their antiauthentic SARS‐CoV‐2 original strain effect in human pulmonary alveolar epithelial cells (HPAEpiCs). GMT value was 2.7 × 10^3^ (Figure 2C), consistent with that in the pseudovirus assay. These results suggest that the rabbit anti‐HR212 sera highly inhibit SARS‐CoV‐2 infection in vitro.

Using the competitive enzyme‐linked immunosorbent assay (ELISA) as previously reported,^15^ we incubated anti‐HR212 sera and HRP‐labeled HR1, and then added them to HR212 that had been precoated on the ELISA plate. It demonstrated that the anti‐HR212 sera could effectively inhibit HR1 and HR212 binding dose‐dependently, and the GMT value was 9.0 × 10^2^ (Figure 2D). To confirm that the HR212 antibodies in the sera were indeed involved in this blocking effect, we pretreatment of the coated HR212 with the sera, followed by washing off unbound sera, and then added HRP‐labeled HR1. It demonstrated that the sera similarly blocked the binding of HR1 to HR212, with a GMT value of 3.1 × 10^2^ (Figure S1A). This result indicates the inhibition of the HR212/HR1 interaction is primarily mediated by antibodies that bind with the coated HR212. In addition, we also found anti‐HR212 sera can inhibit HR2 binding to HR121, with a GMT value of 2.7 × 10^2^ (Figure S1B). Thereafter, we conducted cell‐cell fusion experiments and found that HR212 sera apparently suppressed S protein‐induced cellular membrane fusion (Figure S1C). Viral entry into host cells process is classified as two stages: attachment and membrane fusion. Virus attachment is allowed to occur at a temperature of 4°C, while subsequent membrane fusion processes can be initiated at 37°C. To investigate the specific targeting of membrane fusion by anti‐HR212 serum, we incubated cells with the virus at 4°C for 1 h before adding the serum. As a negative control, the anti‐RBD serum was used to block virus adsorption without affecting membrane fusion. The results demonstrated that after viral attachment onto cell surface, adding anti‐RBD serum (concentration is 10‐fold IC_50_ against the SARS‐CoV‐2 ancestral strain) had minimal effect on blocking viral entry, whereas addition of anti‐HR212 serum (10‐fold IC_50_ against the ancestral strain) still effectively blocked viral entry. This indicates that anti‐HR212 serum does not exert its action during the adsorption stage (Figure S1D,E). We also tested whether similar antibodies were present in the sera of COVID‐19 convalescents, vaccinees (from Sinovac vaccinees) and hybrid immunity. In line with the previous results,^12^ moderate anti‐HR1, anti‐HR2, and anti‐HR12 antibody levels were detected within sera from the above samples (Figure 2E), but none of the sera could prevent the binding of HR1 and HR212, which means the production of less membrane fusion‐targeting nAbs in the above samples (Figure 2D). Therefore, akin to the HR121 protein vaccine previously reported,^12^ the recombinant protein HR212 is also a specific immunogen that can stimulate high levels of nAbs to inhibit membrane fusion, while the viral infection or some currently used vaccines is unable to evoke such nAbs.

Since the HR2 domain is highly conserved among different coronaviruses, the nAbs targeting it should be broad‐spectrum for SARS‐CoV‐2 variants. We therefore examined neutralizing effect of rabbit anti‐HR212 sera against 28 prior and existing SARS‐CoV‐2 variants using pseudovirus assays. As expected, the anti‐HR212 sera effectively inhibited these pseudoviruses entering 293T‐ACE2 cells, with NT_50_ titers ranging from 2.0 × 10^2^ to 3.9 × 10^4^. Encouragingly, the rabbit anti‐HR212 sera could effectively inhibit all the nine tested Omicrion variants entering into 293T‐ACE2 cells, and the NT_50_s against the currently circulating variants, such as BA.2.86 and JN.1 were 4.3 × 10^2^ and 1.1 × 10^3^, respectively. Interestingly, the nAb titers against some variants, such as B.1.351 and C.37, were higher than that against ancestral strain, the reason for it remains unknown and necessitates further exploration (Figure 2F–I and Table S2).

Besides this, we immunized rabbits with RBD protein (from ancestral strain) or HIV‐1 Tat protein (a protein from HIV‐1 served as an unrelated control) and examined the neutralizing activity of these sera using pseudovirus assays. The results are consistent with expectations, the RBD induced high binding antibodies (bAbs) to itself (Figure S1F), which showed high neutralizing activity against the ancestral strain (Figure S1G) but demonstrated almost none of neutralizing activity against some other variants, such as the tested Omicrion BQ.1 and EG.5 (Figure S1H,I). The results further indicate that compared with nAbs induced by RBD, the nAbs evoked by HR212 have broad‐spectrum anti‐SARS‐CoV‐2 activities.

**FIGURE 3:**
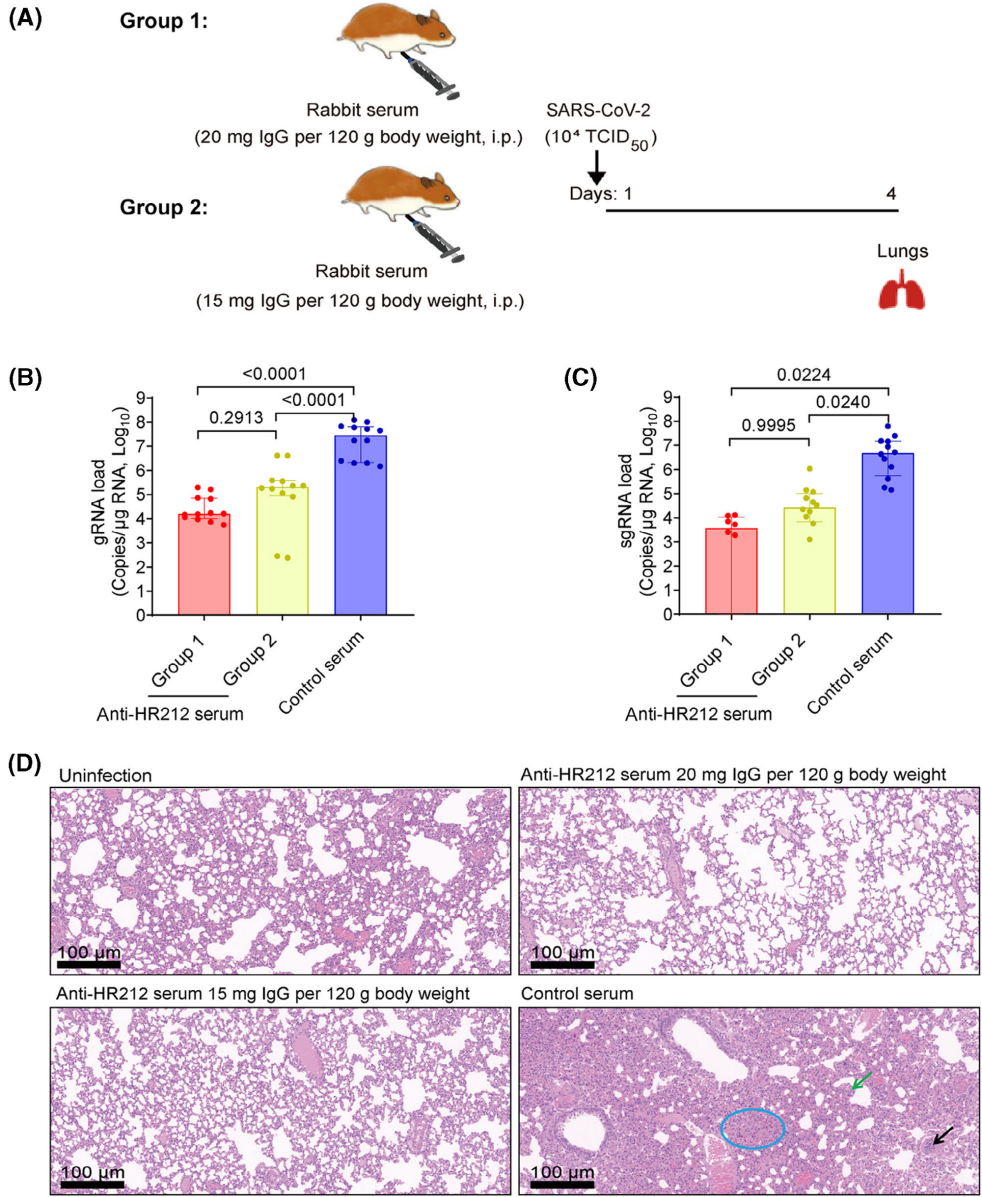
Rabbit anti‐HR212 sera protected Syrian golden hamsters from SARS‐CoV‐2 ancestral strain infection. (A). Schematic diagram of passive immunizing rabbit anti‐HR212 sera into Syrian golden hamsters. Rabbit anti‐HR212 sera were transferred intraperitoneally to two groups of hamsters at the dose of 20 mg lgG per 120 g body weight or 15 mg lgG per 120 g body weight 24 h before infection (*n* = 12). Quantitative PCR was used to analyze SARS‐CoV‐2 genomic RNAs (gRNAs) (B), and subgenomic RNAs (sgRNAs) (C). Pathological changes in lung tissues were evaluated using HE staining (D). Each point represents an individual, and representative pathologic figures were selected randomly. The thickened alveolar wall (green arrow), inflammatory cell infiltration (black arrow), and unrecognizable architecture (blue circle) were labeled. Data are presented as median ± interquartile range and differences between each group are determined by one‐way ANOVA. TCID_50_, tissue culture infective dose.

We then transferred intraperitoneally the pooled rabbit anti‐HR212 sera into Syrian golden hamsters and assessed lung pathological alterations and viral loads after SARS‐CoV‐2 ancestral strain challenge at the higher titer (TCID_50_ = 1.0 × 10^4^) (Figure 3A). The anti‐HR212 sera significantly reduced pulmonary viral loads in the infected hamsters, with viral genomic RNAs (gRNAs, an indicator of the total viral particles) declined 2.1 and 3.2 Logs of low‐ and high‐dose groups, respectively; while subgenomic RNAs (sgRNAs, an indicator of the replicating viral particles) dropped 2.2 and 3.1 Logs of low‐ and high‐dose groups, respectively (Figure 3B,C). Encouragingly, no obvious pathological changes were observed in the two anti‐HR212 serum administration groups, while severe lung lesions were observed from control serum group, like alveolar wall thickening, infiltration of inflammatory cells, separation of bronchial mucosa, and unrecognizable architecture (Figure 3D). Thus, passive transfer of rabbit anti‐HR212 sera could restrict viral replication in vivo.

### 2.3 HR212 induced HR2‐specific cellular and humoral immunity within BALB/c mice

**FIGURE 4:**
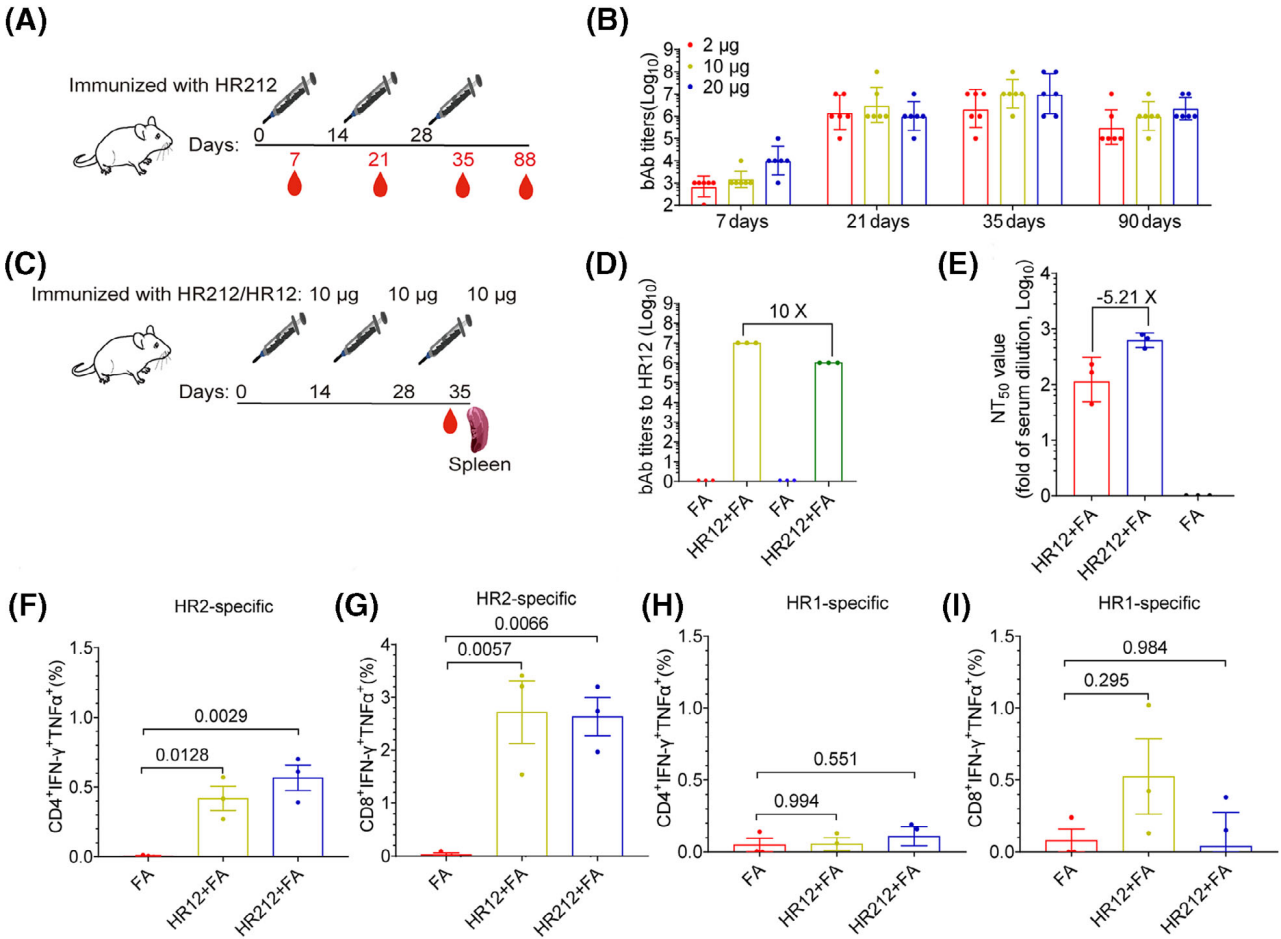
HR212 induced humoral and cell‐mediated immune responses in BALB/c mice. (A) Schematic diagram of BALB/c mice immunized with HR212 at 2‐week intervals. Three groups of mice were immunized with different doses of HR212 in Freund's adjuvant (FA), and another two groups were injected with FA and PBS as negative controls. In each group, *n* = 6. (B) End‐point titers of HR212 binding antibodies (bAbs). No bAbs were detected in the other two groups of mice injected with Freund's adjuvant and PBS. (C) Schematic diagram of BALB/c mice immunized with HR212 at 2‐week intervals. Three groups of mice were immunized with different doses of HR212 or HR12 in Freund's adjuvant (FA), and another group was injected with FA as negative controls. In each group, *n* = 3. (D) Titers of bAbs to HR12 at 7 days after the third immunization. *n* = 3 (E) The titers of neutralizing antibodies (nAbs) against SARS‐CoV‐2 ancestral strain in mice sera. *n* = 3. (F–I) The HR2 and HR1‐specific cellular immunity induced by HR12 or HR212 immunization in mice. *n* = 3. In (B), (D), and (E), data are presented as geometric mean ± geometric SD, in (F–I) data are presented as median ± interquartile range. The differences between each group are determined by one‐way ANOVA.

For evaluating antibody generation, BALB/c mice in 3 groups were subcutaneously injected three times with 2, 10, and 20 µg of HR212 in Freund's adjuvant every 2 weeks (Figure 4A). In each immunization, no significant difference was observed in IgG production in diffident groups. The initial dose induced a low level of HR212‐specific IgGs (GMTs ranged from 6.3 × 10^2^ to 1.0 × 10^4^) and the antibody titers apparently elevated following the second dose (GMTs, 1.0 × 10^6^ to 3.2 × 10^6^), reached the peak following the third dose (GMTs, 2.5 × 10^6^ to 1.0 × 10^7^) (Figure 4B). Promisingly, the levels of HR212‐specific IgGs remained at high levels 2 months following the third dose (GMTs ranged from 4.0 × 10^5^ to 2.2 × 10^6^) (Figure 4B).

To further assess the effect of immunization interval on antibody production, BALB/c mice were subjected to immunization using 10 µg HR212 every 3 weeks (Figure S2A). According to the results, the antibody titer after the third immunization was approximately 2.7‐fold higher (GMT = 6.8 × 10^6^) than that produced in 2‐week intervals (Figure S2B). We then collected their spleen cells after the third immunization and cocultured the spleen cells with HR212 and found that the cells secreting HR212‐specific lgGs remarkably increased (Figure S2C,D). We also cocultured them with a 15‐amino acid overlapping peptide pool covering the entire HR2 sequence and observed that the interferon‐γ (IFNγ)‐secreting cells were greatly enhanced (Figure S2E,F).

Like HR121 immunization, HR212 immunization produces high HR12 antibodies. The antibodies induced by HR12 also have a weak neutralizing activity,^12^ it is difficult to determine which antibody is responsible for the neutralizing activity in this study. To solve this problem, we immunized mice with HR212 and HR12 respectively. Given the minimal difference in antibody titers between the 3‐week interval and 2‐week interval groups, to shorten the immunization cycle, we opted for a 2‐week interval (Figure 4C). We collected serum samples and spleen cells from mice receiving immunization 7 days following the third dose. Anti‐HR12 sera contained high bAbs target HR12, but it induced poor nAbs. The bAb titers to HR12 were 10 folds higher than that in anti‐HR212 sera (Figure 4D), but the nAb titers were 5.21 folds lower than those in HR212 sera (Figure 4E). This result implies that the HR12 bAbs have minimal influence on SARS‐CoV‐2 neutralization, and that HR212 immunization‐induced nAbs may mainly target the conformational epitopes of the three HR2s.

Subsequently, the spleen cells were stimulated with HR2 or HR1 peptide pools, followed by measurement of T cell responses using flow cytometry (Figure S3). The results showed that HR2 peptide pools invoked strong CD8+ T cell responses, manifested as an increase in the number of IFNγ and TNFα double positive cells, whereas the response of CD4+ T cells was limited (Figure 4F,G). The HR1 peptide pools did not induce significant responses of CD4+ T or CD8+ T cells (Figure 4H,I). Similarly, HR12 immunization also exhibited an HR2‐specific CD8+ T cell response, this may be attributed to the concealment of the HR2 trimer within the 6‐HB structure.

Together, these results suggest that a strong HR2‐specific T‐cell and HR212‐specific B‐cell regulated immunity was evoked within HR212‐immunized mice.

### 2.4 Immunization with HR212 prevented Syrian golden hamsters against pulmonary lesions after SARS‐CoV‐2 ancestral strain infection

**FIGURE 5:**
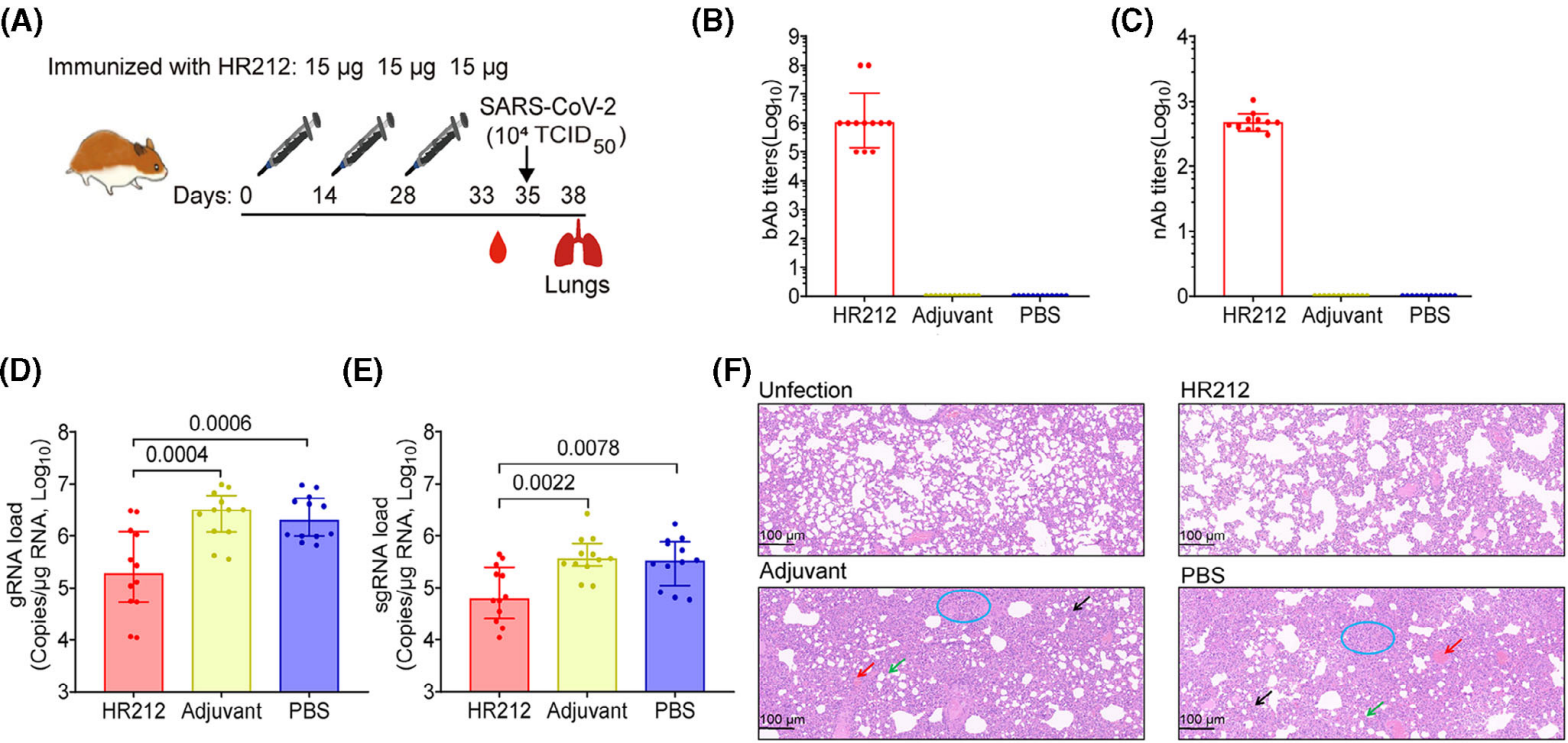
Immunization with HR212 provided effective protection in Syrian golden hamsters from pulmonary lesions against SARS‐CoV‐2 ancestral strain infection. (A) Schematic diagram of Syrian golden hamsters immunized with HR212. one group of hamsters were injected with HR212 in Freund's adjuvant (FA), and another two groups were injected with FA and PBS as negative controls (*n* = 12 in each group). SARS‐CoV‐2 ancestral strain (TCID_50_ = 10^4^) challenge was carried out 7 days after the last vaccination. (B) End‐point titers of HR212 binding antibodies (bAbs). (C) The titers of neutralizing antibodies (nAbs) against SARS‐CoV‐2 ancestral strain in hamster sera immunized with HR212. (D and E) Quantitative PCR was used to analyze SARS‐CoV‐2 genomic RNAs (gRNAs) (D), and subgenomic RNAs (sgRNAs) (E). (F) HE staining in lung tissue. Each point represents an individual, and representative pathologic figures were selected randomly. The infiltration of lymphocytes and macrophages (black arrow), thickening of alveolar wall (green arrow), unrecognizable architecture (blue circle), and pulmonary hemorrhage (red arrow) were labeled. In (B) and (C), data are presented as geometric mean ± geometric SD. In (D) and (E), data are presented as median ± interquartile range, and differences between each group are determined by one‐way ANOVA.

Thereafter, we immunized Syrian golden hamsters with 15 µg of HR212 in Freund's adjuvant three times every 2 weeks (Figure 5A). A high titer of HR212‐specific IgGs (GMT = 1.2 × 10^6^) was evoked in HR212‐immunized hamsters upon the third immunization (Figure 5B) and the neutralizing titer was 4.7 × 10^2^ (Figure 5C). After SARS‐CoV‐2 ancestral strain (TCID_50_ = 1.0 × 10^4^) challenges, the pulmonary viral loads in the HR212‐immunized hamsters were significantly reduced (Figure 5D,E); compared with the adjuvant and PBS control groups, the median value of gRNAs decreased by more than 10‐fold (Figure 5D), and the similar degree of decline was observed in sgRNAs (Figure 5E). Although the HR212‐immunized hamsters had weak protection from viral replication, not effective as that of the reported HR121 vaccine,^12^ but compared with the uninfected control group, the HR212‐immunized group did not show significant lesions, only a moderate thickening of the alveolar wall (Figure 5F). However, in the two control groups, lung tissues displayed representative viral interstitial pneumonia characteristics, like macrophage and lymphocyte infiltration, thickening of alveolar wall, unrecognizable architecture, and pulmonary hemorrhage (Figure 5F). Therefore, immunization with HR212 reduced the viral loads and dramatically alleviated the lung pathological lesions within the SARS‐CoV‐2 ancestral strain‐infected hamsters.

### 2.5 Immunization with HR212 provided effective protection for Syrian golden hamsters from infection with Omicron BA.2 variant

**FIGURE 6:**
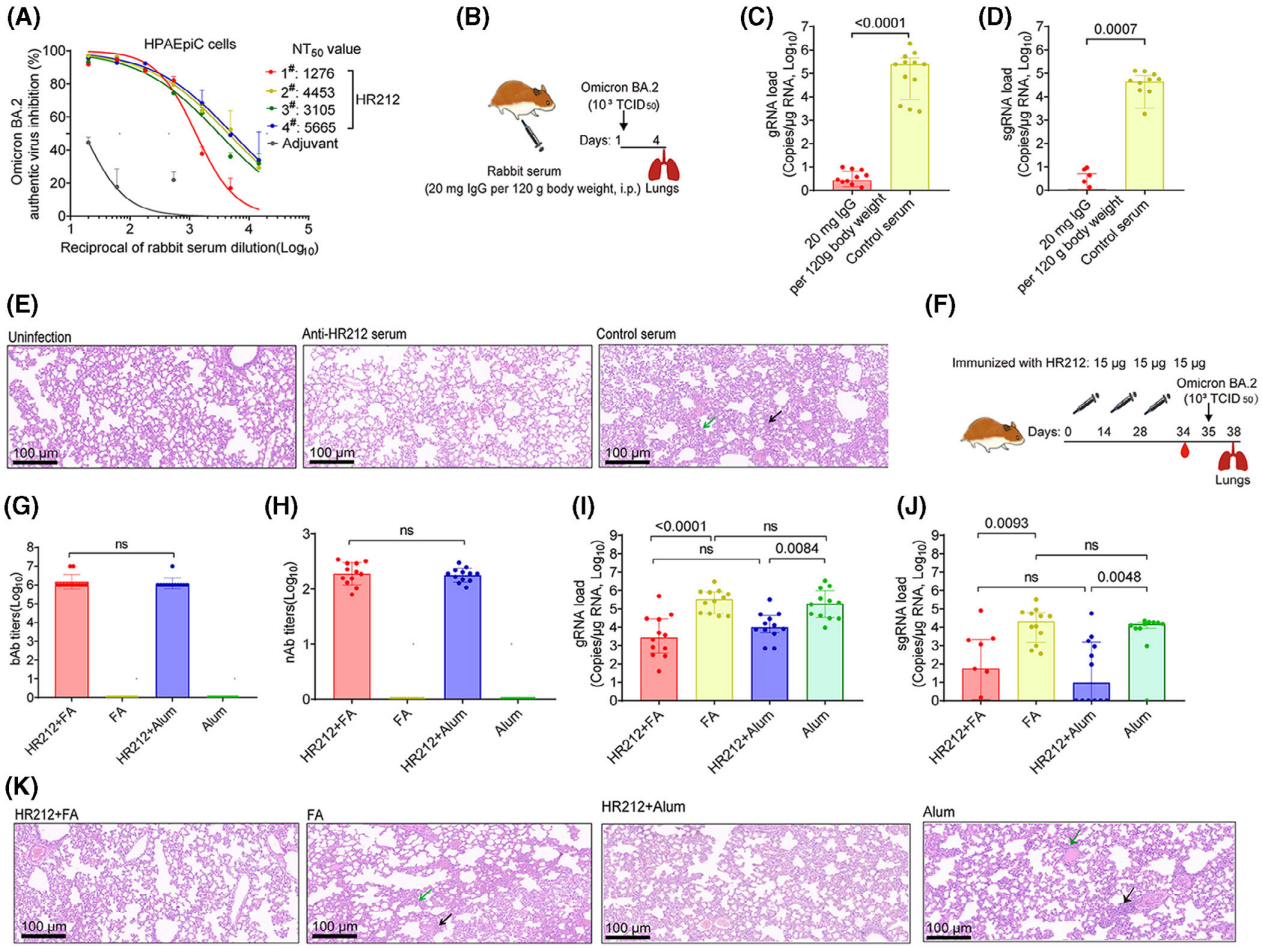
Immunization with HR212 effectively protected Syrian golden hamsters from Omicron BA.2 variant infection. (A) Titers of nAbs against authentic Omicron BA.2 variant in rabbits anti‐HR212 sera. (B–E) Rabbit anti‐HR212 sera could protect hamsters from Omicron BA.2 variant infection (*n* = 12). Schematic diagram of passive immunization of hamsters with rabbit anti‐HR212 sera. Rabbit sera immunized with HR212 were transferred intraperitoneally to hamsters at the dose of 20 mg lgG per 120 g body weight 24 h before infection (B). Quantitative PCR was used to analyze Omicron BA.2 genomic RNAs (gRNAs) Omicron BA.2 (TCID_50_ = 10^3^) challenge was carried out 1 day after vaccination (C), and subgenomic RNAs (sgRNAs) (D), and HE staining analysis of lung tissues (E). (F) Schematic diagram of hamsters immunized (*n* = 12 in each group) with HR212 in Freund's adjuvant (FA) or aluminum adjuvant (alum). Omicron BA.2 (TCID_50_ = 10^3^) challenge was carried out 7 days after the last vaccination (G) End‐point titers of HR212 binding antibodies (bAbs). (H) The titers of neutralizing antibodies (nAbs) against Omicron BA.2 viruses in hamster sera immunized with HR212. (I and J) Quantitative PCR was used to analyze Omicron BA.2 genomic RNAs (gRNAs) (I), subgenomic RNAs (sgRNAs) (J). (K) HE staining in lung tissues. In (C), (D), (G), (H), (I), and (J), each point represents an individual. In (E) and (K), the infiltration of lymphocytes and macrophages (black arrow), and thickening of alveolar wall (green arrow) were labeled, and representative figures were selected randomly. In (A), (G), and (H) data are presented as geometric mean ± geometric SD. In (C), (D), (I), and (J), data are presented as median ± interquartile range, and differences between each group are determined by one‐way ANOVA.

Currently, Omicron sublineages are the predominant SARS‐CoV‐2 variants. Rabbit anti‐HR212 sera showed potently cross‐neutralization effect on several Omicron variants from pseudovirus assays (Figure 2F–I). As most of currently circulating Omicron variants are originated from BA.2 strain (https://covid.cdc.gov/covid‐data‐tracker/#variant‐proportions), we further tested in vitro inhibition on Omicron BA.2 variant within HPAEpiCs. Similar to its inhibitory activity against Omicron BA.2 pseudovirus (NT_50 _= 9.3 × 10^2^) (Figure 2F), the anti‐HR212 sera effectively blocked Omicron BA.2 variant replication in the HPAEpiCs, with NT_50_ values ranging from 1.3 × 10^3^ to 5.7 × 10^3^ among the sera of the four HR212‐immunized rabbits (Figure 6A).

Subsequently, this study analyzed in vivo protection effect of rabbit anti‐HR212 sera against the Omicron BA.2 variant. Given the observation that HR212 provided a weak protection from the viral replication upon the SARS‐CoV‐2 ancestral strain challenges (Figures 3A–C and 5D,E), which may be related to a high titer (TCID_50_ = 1.0 × 10^4^) of viral inoculation, we then decreased the dose of Omicron BA.2 variant to a 10‐fold lower titer (TCID_50_ = 1.0 × 10^3^) than that of ancestral strain in the following challenge assays. After intraperitoneally injecting the anti‐HR212 sera into Syrian golden hamsters (Figure 6B), here, we observed that the anti‐HR212 sera almost completely protected the hamsters from the BA.2 infection. In the group of anti‐HR212 sera administration, extremely low levels of viral gRNAs and sgRNAs were detected (Figure 6C,D), and nonsignificant pathological changes were observed (Figure 6E). Furthermore, the same approach was conducted for evaluating protection of anti‐RBD and anti‐HIV‐1 Tat sera in hamsters following BA.2 challenge. It was observed that neither anti‐RBD nor anti‐Tat sera exhibited a significant protective role in controlling viral replication (Figure S4A–C), thus the subsequent pathological protection was not conducted.

For assessing protection efficacy of HR212 vaccination in vivo, this study Syrian golden hamsters from two groups using 15 µg of HR212 three times in 2‐week intervals, including one group receiving HR212 vaccination in Freund's adjuvant that was just used as reference, whereas the other group undergoing HR212 vaccination in aluminum adjuvant that was used as reference only (Figure 6F). Seven days after the third immunization, both HR212 formulated with Freund's adjuvant and HR212 formulated with aluminum adjuvant induced similarly high titers of HR212‐bAbs, with GMT values of 1.4 × 10^6^ and 1.2 × 10^6^, respectively (Figure 6G). nAbs for SARS‐CoV‐2 ancestral strain elicited in both groups also exhibited no significant difference, with the GMT values at 1.9 × 10^2^ and 1.8 × 10^2^, respectively (Figure 6H). After Omicron BA.2 variant (TCID_50_ = 1.0 × 10^3^) challenge, both the hamsters immunized with HR212 in Freund's adjuvant and the hamsters immunized with HR212 in aluminum adjuvant showed remarkable decreases in pulmonary gRNA loads, with median values declining 2.1 Logs and 1.3 Logs, respectively; compared with those in each adjuvant control (Figure 6I). Importantly, as the viral sgRNAs were considered, aluminum adjuvant demonstrated a similarly effective protection as Freund's adjuvant. The sgRNA loads within lung tissues in HR212‐immunized hamsters decreased 3.2 Logs in aluminum adjuvant group, and 2.6 Logs in Freund's adjuvant group, separately, and difference was not of statistical significance (Figure 6J). Following H&E staining for lung tissues, obvious pathological lesions could not be detected from HR212 plus aluminum adjuvant group and HR212 plus Freund's adjuvant group, while in the adjuvant controls, slight‐to‐moderate interstitial pneumonia, such as macrophage and lymphocyte infiltration, and thickening of alveolar wall could be observed (Figure 6K). Thus, compared with Freund's adjuvant, clinical‐grade aluminum adjuvant could evoke the similarly effective HR212‐specific antibody response to inhibit viral replication and protect pulmonary lesions from Omicron BA.2 variant infection.

Collectively, passive immunization with rabbit anti‐HR212 sera or vaccination with HR212 protein effectively protected Syrian golden hamsters from Omicron BA.2 variant infections.

## 3 DISCUSSION

The continuously emerging Omicron sublineages, with multiple mutation sites located in the RBD domain of S1 subunit, have decreased neutralization by infection‐ and vaccine‐induced immune responses and have increased transmission and re‐infection abilities.^16^, ^17^, ^18^ Thus, this calls for other more conserved targets apart from the S1 subunit for novel vaccine designs.

The S2 subunit exerts a relatively weaker selective pressure from the host, and consequently presents with fewer mutations in the emerging variants than the S1 subunit.^16^ However, fewer nAbs targeting S2 subunit have been isolated following SARS‐CoV‐2 infection or immunization with bacterially expressed recombinant S2 protein,^19^, ^20^, ^21^ suggesting that the naive conformation of the S2 subunit contains few neutralizing epitopes that are barely recognizable by the host immune system.

During membrane fusion, the S2 subunit may experience a transient conformational rearrangement from “prefusion” to “fusion intermediate,” wherein the three HR1s form an exposed inner trimer, whereas the three free HR2s tandemly link with the trimer.^22^, ^23^ This model is widely accepted for most of class I envelope viruses, and based on it, a large number of HR2 peptides derived from these viral species like HIV‐1, coronaviruses and influenza virus, have been developed as potent fusion inhibitors, these peptides can inhibit the binding of HR1s with HR2s in the “fusion intermediate,” and consequently suppress “6‐HB postfusion” structure generation.^9^ However, for COVID‐19 vaccinated or convalescent individuals, few HR1‐ and HR2‐targeting nAbs were isolated,^13^, ^21^ we herein also did not detect such nAbs, which may be because the “fusion intermediate” conformation exists for only several minutes during membrane fusion,^24^ implying the importance of constructing a stable immunogen to mimic its conformation in vaccine designs.

In the previous study, the recombinant protein vaccine HR121 was prepared, which can mimic HR1 domain conformation within “fusion intermediate” in S2 subunit and induce highly and broadly nAbs for SARS‐CoV‐2 as well as the corresponding variants. Besides, “6‐HB postfusion” structure induced very weak nAbs against SARS‐CoV‐2.^12^ Therefore, “fusion intermediate” in S2 subunit is the promising target for designing vaccines. Here, using the rationally designed recombinant protein HR212 which mimics the conformation of the three HR2s in the “fusion intermediate,” we demonstrated that the HR2 domain can also induce broadly nAbs against SARS‐CoV‐2 along with the associated variants. Compared with HR1 domain, HR2 domain shows the higher conservation degree. The SARS‐CoV‐2 ancestral strain shares 92.6% identity in amino acid sequence with SARS‐CoV for the HR1, whereas 100% for the HR2.^9^ Moreover, when the current Omicron sublineages were considered, they share several mutations of HR1 domain, such as Q954H/N969K/L981F in Omicron BA.1 sublineages, and Q954H/N969K in the following other Omicron sublineages; but no mutations in the HR2 domain,^25^ which suggests that HR212 may induce more broadly nAbs against Omicron sublineages, even against SARS‐CoV or more associated coronaviruses.

In past decades, several mimics of “fusion intermediate” from additional class I enveloped viruses were constructed, such as 5‐helix,^26^ N35CCG‐N13,^27^ (CCIZN36)3/(CCIPN36)3,^28^ N46FdFc from HIV‐1,^29^ and 5‐helix from human respiratory syncytial virus,^30^ they can mimic the conformations of HR1 domains under “fusion intermediate” state in these viruses while inducing nAbs in vivo. However, few HR2‐targeting immunogens are obtained at present. Recently, a study demonstrated that the truncated HR212 within S2 subunit in SARS‐CoV‐2 could trigger highly and broad‐spectrum nAbs against SARS‐CoV‐2 as well as the corresponding variants.^31^ Meanwhile, antibody‐dependent cytotoxicity (ADCC) induced by NK cells can facilitate SARS‐CoV‐2 control.^32^, ^33^ As there are many mutation sites within S1 subunit, especially in the RBD domain, the ADCC effect of antibodies induced by S1 subunits may be compromised by mutations.^32^ In contrast, antibodies targeting the S2 subunit did not showed significant ADCC evasion,^32^ therefore, the antibodies induced by HR212 may benefit from ADCC and warrant further investigation. These studies, together with ours, suggest that the HR212 design approach may serve as a starting point to develop broad‐spectrum nAbs and vaccines for SARS‐CoV‐2 and certain class I enveloped viruses.

It should be noted that the nAbs induced by HR212 were relatively weak in the present study, providing less protection from SARS‐CoV‐2 ancestral strain as well as Omicron BA.2 variant infections than those induced by HR121.^12^ Also, the HR212 crystal structure was not obtained, whose reasons is not unknown but may be due to the free conformation of the three HR2s in HR212 trimer, which may have also reduced the efficiency of antigenic epitopes in the HR2s. Therefore, in the aim of developing the HR212‐based vaccines, further optimization of HR212 is needed to improve its immunogenicity, such as covalent stabilization at the N‐terminals of the three free HR2s through 3 interchain disulfide bonds formed between cysteine pairs,^28^ or conjugation HR212 with some immunopotentiator.^29^ Typically, non‐human primates (NHP) model is the suitable model in evaluating SARS‐CoV‐2 infection as well as COVID‐19 vaccines.^34^ However, antiviral activity of HR212 was relatively weak in our expensive NHP experiment. Our previous work has shown that the HR121 vaccine targeting membrane fusion can prevent rhesus monkeys against SARS‐CoV‐2 infection.^12^ We will optimize the designs of HR212 to improve its immunogenicity and the subsequently evoked antibody activity for the NHP experiment. Meanwhile, in future studies, the effectiveness of optimized HR212 in aluminum adjuvant or other better adjuvants approved for human use should be assessed in vivo. At last, we noticed that HR2 domain of SARS‐CoV‐2 contain two glycosylation sites.^35^ Considering that HR212 expressed in *E. coli* does not have glycans, further investigation and comparison of the immunogenicity of prokaryotic and eukaryotic HR212s is warranted.

Collectively, HR212 mimics HR2 domain of S2 subunit and induce broadly nAbs to resist SARS‐CoV‐2 together with the corresponding variants. Thus, the conserved HR2 domain is the novel and potential target used to design vaccines.

## 4 MATERIALS AND METHODS

### 4.1 Human sera

Ten sera were collected in subjects vaccinated with SARS‐CoV‐2, meanwhile, one serum sample was obtained from a normal unvaccinated subject. Inactivated SARS‐CoV‐2 vaccines (Sinovac Biotech, Beijing, China) were injected in the subjects at two doses in 1–2 months. All subjects provided informed consent. Twenty serum samples from COVID‐19 convalescent individuals and 10 from SARS‐CoV‐2 vaccinated subjects followed SARS‐CoV‐2 infection were obtained from Yunnan Provincial Infectious Disease Hospital, China. Each convalescent participant achieved recovery from COVID‐19 within 1–2 months. Informed consents were obtained as well.

### 4.2 Viruses, cells, and animals

Ancestral SARS‐CoV‐2 strain (China National Microbiology Data Center (NMDC) with No. NMDCN0000HUI),^36^ and Omicron BA.2 variant (NMDC with No. NMDC60046377) were propagated at Kunming National High‐level Biosafety Research Center as previously reported.^12^ The 6th to 8th passages of both isolates were used in this study. VSV‐G pseudovirus G*ΔG‐VSV‐Rluc) was obtained from Professor Geng‐Fu Xiao, Wuhan Institute of Virology, Chinese Academy of Sciences. 293T‐ACE2 cell was purchased from Yesean Company (Shanghai, China), and human alveolar epithelial cells (HPAEpiCs) were obtained in ScienCell Research Laboratory (San Diego, CA, USA). All the cells were cultivated within DMEM medium that contained 10% fetal bovine serum (Gibco). All the experimental animals used in this study, including BALB/c mice rabbits, and Syrian golden hamsters, were purchased from Beierji Biotech (Kunming, China).

### 4.3 Plasmid establishment, protein purification, and structure modeling

Expression vectors were constructed, and proteins were purified according to previous description.^12^ In brief, genes encoding HR212 were synthesized and inserted in *E. coli* expression vectors pET‐30a and pGEX‐6P‐1, locating in *Eco*RI and *Xho*I enzyme sites, respectively.

The recombinant protein HR212, HR12, and HIV‐1 Tat (an unrelated protein control) was induced expression within *E. coli* BL21 (DE3) cells. Later, 1 mM IPTG was added to induce bacteria at 20°C for a 12‐h period prior to harvest through centrifugation. All collected bacteria underwent ultrasonic lysis within PBS and centrifugation to remove sediment. The Ni‐Sepharose 6 FF affinity column for expression in pET‐30a vector and glutathione agarose 4B affinity gel was used to purify target proteins for expression in pGEX‐6P‐1 vector, respectively (GE Healthcare). The structure of HR212 was predicted by Alpha Fold software. The prediction results were compared with SARS‐CoV‐2 6‐HB crystal structure homology (PDB:6LXT) and graphics were generated by PyMOL program.

### 4.4 GST‐pull down assay

The excessive HR1 and HR2 within supernatant of classified bacteria were added into glutathione agarose 4B affinity gel that contained GST‐HR212. Besides, HR1 or HR2 was added into blank glutathione Sepharose 4B affinity gel, with glutathione Sepharose 4B affinity gel that contained GST‐HR212 being the negative control. Thereafter, this resultant mixed sample underwent 30 min of incubation at room temperature. Then, through 5 min of 1000×*g* centrifugation for removing supernatant, the gel was rinsed three times using PBS prior to 10% SDS‐PAGE for separation.

### 4.5 Surface plasmon resonance assay

Biacore 3000 instrument (GE Healthcare) was employed to determine whether HR212 bound to HR1 through conducting surface plasmon resonance. HR212 is fixed to flow cell in the CM5 sensor. HR1 proteins (12.5, 25, 50, 100, 200 nM) were added for crossing the chip. Combined analysis was performed at 25°C, followed by collection of separate data within 10 min. Biacore evaluation software version 4 was utilized to acquire kinetic parameters.

### 4.6 Circular diachronic spectrum

Record the CD spectrum using a CD spectrometer (Applied Photophysics). 1 µM HR212 and 1 µM HR1 dissolved in PBS. Using a cuvette with an optical path of 0.1 cm, the wavelength spectra of 200–250 nm were recorded in 1‐nM steps and 1‐nM bandwidth under 20°C. Solvent blank PBS was subtracted to correct spectra.

### 4.7 bAb detection

The bAbs against HR1, HR2, HR12, HR212 and RBD within sera were detected through the sandwich ELISA. In brief, 1 µg/mL HR1, HR2, HR12, HR212 or RBD protein within the coating buffer (consisting of 15 mM Na_2_CO_3_, 35 mM NaHCO_3_, pH 9.6) was introduced in 96‐well polystyrene plate to incubate overnight. Later, coating buffer was removed, plates were rinsed by PBS that contained 0.05% Tween‐20 (PBST) thrice, followed by 2 h of blocking using 5% BSA‐containing PBST under 37°C. After washing three times, the plate was added continuously diluted serum samples for 2 h of incubation under 37°C. Following rinsing, secondary antibody linked with HRP was introduced and incubated at 37°C for 1 h. Every well was introduced the OPD substrate following rinsing, and 2 mM H_2_SO_4_ was added to terminate the reaction_._ Optical density (OD) value in each well was measured at 490/630 nm using the ELISA reader. Serum sample endpoints were identified as reciprocal of final dilution with OD values above 2.1 times the mean background value.

### 4.8 Inhibition of binding between HR1 and HR212

We carried out competitive ELISA for detecting serum antibodies that blocked the binding of HR1 to HR212 or HR2 to HR121. 1 µg/mL HR121 or. HR212 protein was applied to ELISA plate overnight under 4°C. Following removing coating buffer, plates were rinsed by PBST prior to 2 h of incubation using blocking buffer under 37°C. After washing thrice using PBST, serum samples from humans or rabbits were threefold diluted with PBS, followed by 30 min of preincubation using 100 ng/mL HR2 or HR1‐labeled HRP under 20°C. This mixed sample was then introduced into every well to incubate for a 1‐h duration under 37°C. In another way, the plate was coated with HR212 and washed with PBST similarly, then the diluted serum samples were added. After washing, HR1 labeled HRP was added. The plate was cleaned, OPD substrate was added, and OD value in each well was measured at 490/630 nm.

### 4.9 SARS‐CoV‐2 pseudovirus neutralization assay

This study obtained VSV‐G pseudotyped virus (G*ΔG‐VSV‐Rluc) from Prof. Geng‐Fu Xiao (Wuhan Institute of Virology, CAS). A total of 29 VSV‐based SARS‐CoV‐2 pseudoviruses (including one ancestral strain together with 28 variants) were generated according to previous description.^12^ The multiple mutations in each spike gene were listed in Table S1, including the ancestral strain, S477N, E484K, A222 V, N439K, K417N, D614G, D839Y, B.1.617, B.1.617.1, B.1.617.2.V2, B.1.429, B.1.525, B.1.526, B.1.1.7, B.1.351, B.1.1.28, B.1.617.2, C.37, and B.1.621; as well as Omicron BA.1, BA.2, BA.3, BA.4/5, BQ.1, XBB 2.3, EG.5, BA.2.86, and JN.1. For determining serum neutralizing effect, we seeded 100 µL 293T‐ACE2 cells (1 × 10^4^/well) into 96‐well plate. On following day, serum samples from HR212‐immunized rabbits were added into another 96‐well plate at 60 µL in threefold dilutions. Thereafter, we introduced SARS‐CoV‐2 pseudovirus (60 µL, M.O.I. = 0.1) into diluted serum samples for 1 h of incubation under 37°C. Subsequently, pseudovirus‐serum mixture (100 µL) was added into 293T‐ACE2 cells for 24‐h culture under 37°C. Renilla luciferase activity of each well was determined using Renilla luciferase assay kit (Promega).

### 4.10 SARS‐CoV‐2 authentic virus neutralization assay

1.6 × 10^5^ of HPAEpiCs (200 µL/well) were inoculated into 48‐well plates overnight. On following day, we added 100 µL serum samples collected in HR212 immunized animals into another 48‐well plate in twofold dilution, followed by addition of SARS‐CoV‐2 ancestral or Omicron BA.2 (M.O.I. = 1) (100 µL) to diluted serum samples for 1‐h incubation at 37°C. Subsequently, after removing culture medium, virus‐serum mixture was added to culture HPAEpiCs for an additional 1‐h duration under 37°C, and this mixed sample was rinsed thrice by PBS. Thereafter, freshly prepared medium that contained diluted serum samples was introduced for 48 h of culture under 37°C, later, supernatant was harvested to extract viral RNA (Roche Diagnostics), and the viral load (viral genome RNAs) measurement. The detection limit of viral load was 10 copies/µg RNA.

### 4.11 Animal immunization

Animals including BALB/c mice, rabbits, and Syrian golden hamsters were immunized subcutaneously through the prime‐boost‐reboost method. In the Freund's adjuvant vaccination, we introduced complete Freund's adjuvant (Sigma–Aldrich) into prime immunization, whereas incomplete Freund's adjuvant (Sigma–Aldrich) into 2 boost immunizations. For aluminum adjuvant vaccination, Alhydrogel® adjuvant 2% (Invivogen, San Diego, CA, USA) was used in the three times of immunizations. No changes in animal behavior, body weight, fur ruffling, or appetite were observed during the different HR212 immunizations, and all the animals survived during viral challenges.

On day 0, rabbits (6 months old), rabbits were injected 100 µg of HR212, RBD or HIV‐1 Tat protein, followed by injection of 150 µg of proteins on the 21st and 42nd days. Remaining rabbits were given injection of adjuvant at the same amount for controls. At 7 days following the third immunization, we harvested sera.

For 6‐week‐old BALB/c mice, mice in three groups were given injection of 2, 10, or 20 µg of HR212 every 14 days. Mice in two groups were given adjuvant or PBS at the same amount as controls. At 7 days after every immunization as well as 90 days after third immunization, sera were harvested. To detect the effect of immunization interval, mice in the other group were given injection using 10 µg HR212 or HR12 every 21 days.

For Syrian golden hamsters (8 weeks old), the hamsters were divided into four groups: HR212 plus Freund's adjuvant, Freund's adjuvant control, HR212 plus aluminum adjuvant, or aluminum adjuvant control group. In each group, 12 hamsters were inoculated 15 µg of HR212 (or PBS) plus adjuvant every 14 days. Seven days post‐third immunization, serum samples were collected, and intranasal infection was performed with 10^3^ TCID_50_ Omicron BA.2 variant or 10^4^ TCID_50_ SARS‐CoV‐2 ancestral strain. Euthanasia and dissection of hamsters were carried out after 72 h of infection.

### 4.12 Passive immunization and infection

Rabbit sera immunized with HR212, RBD or HIV‐1 Tat protein was mixed and transferred intraperitoneally to two groups of hamsters at the doses of 3 mL serum /120 g body weight and 4 mL serum/120 g body weight, respectively. Approximately 5 mg IgGs were obtained from 1 mL serum.^12^ Same number of hamsters injected with adjuvant‐immunized rabbit sera were utilized to be controls. At 24 h posttransfer, each hamster was intranasally infected with 10^4^ TCID_50_ SARS‐CoV‐2 ancestral strain or 10^3^ TCID_50_ Omicron BA.2 variant. At 72 h postinfection, hamster euthanasia and dissection were completed.

### 4.13 Viral load measurement

Lung tissue RNA was extracted with Trizol (Takara), while its content was determined with nanodrop 2000 (Thermo Fisher Scientific). A Roche kit was utilized to extract RNA from culture supernatant. Viral load was detected using real‐time qPCR with Q5 real‐time PCR system (Life Technologies). Primers for detecting gRNA and sgRNA in SARS‐CoV‐2 were described previously.^12^

### 4.14 Histological analysis

After 4% paraformaldehyde fixation, gradient ethanol dehydration, and paraffin embedding, the tissue sample was prepared into paraffin sections for dewaxing, rehydration and hematoxylin eosin staining as previously reported.^12^

### 4.15 Measurement of immune cells in the spleens

Spleens of immunized mice were collected and ground to pieces, then the 70‐mesh cell strainer was used to filter cell suspension to remove tissue debris. Then the cells were purified using red blood cell lysate, followed by resuspension in fresh RPMI‐1640 medium for flow cytometry and ELISPOT.

The mouse IFNγ ELISPOT Kit (Diaclone research) was utilized to assess SARS‐CoV‐2 HR2‐specific cytotoxic T lymphocytes (CTLs). Briefly, multiscreen HTS IP filter plates (Millipore) underwent precoating using anti‐mouse IFNγ, and splenocytes separated in BALB/c mice (1 × 10^6^) were mixed into overlapping 15‐amino acid peptides that covered 1 µg/mL complete HR2 sequence (General Biotech) for a 24‐h duration. Following incubation, each well was cleaned and stained, while the spots were counted with the ELISPOT reader.

For evaluating SARS‐CoV‐2 HR212‐specific humoral response, HR212 (1 µg/mL) was precoated on the multilayer filter plate. After washing for three times, we introduced splenocytes (1 × 10^6^) separated in BALB/c mice and incubated them for a 24‐h duration. Spots were counted according to the method used for IFN‐γ.

To assess the functional of T cells in the spleens, splenocytes (1 × 10^6^) were mixed with the pool of HR1 or HR2 peptides for 24 h, and the brefeldin A was added 4 h before staining. After 30 min of staining using anti‐CD3‐FITC, anti‐CD4‐BV421, and anti‐CD8‐Percy‐cy5.5 antibodies (BD Pharmingen) under ambient temperature, cells were washed by PBS twice, then fixed and permeabilized on ice for a 30 min duration using fixation and permeabilization solution (BD Pharmingen). Following rinsing twice using BD perm/wash buffer (BD Pharmingen), anti‐IFNγ‐PE‐cy7 and anti‐TNFα‐PE (BD Pharmingen) were added to stain cells overnight, followed by sample analysis on a FCM instrument.

### 4.16 SARS‐CoV‐2 pseudovirus generation

We performed the generation, package, and titration of VSV‐based SARS‐CoV‐2 pseudoviruses as described previously.^12^ Briefly, according to the pcDNA3.1–SARS‐CoV‐2–SΔ18 vector, seven plasmids containing mutations including S477N, E484K, A222 V, N439K, K417N, D614G, and D839Y were constructed by PCR assays. Other 21 plasmids with multiple mutations in the spike genes included B.1.617, B.1.617.1, B.1.617.2.V2, B.1.429, B.1.525, B.1.526, B.1.1.7, B.1.351, B.1.1.28, B.1.617.2, C.37, B.1.621, B.1.1.529 (Omicron BA.1), Omicron BA.2, Omicron BA.3, Omicron BA.4/5, Omicron BQ.1, Omicron XBB.2.3, and Omicron EG.5, Omicron BA.2.86, and Omicron JN.1. They were synthesized with the corresponding gene sequences (Table S1). SARS‐CoV‐2 pseudoviruses were packaged in 293T cells and titrated within 293T‐ACE2 cells. The NT_50_ and GMT values in the serum were calculated.

### 4.17 Cell fusion

To evaluate the inhibition ability of HR212 serum, the 293T cells were prepared by transfecting pcDNA3.1 vector that encoded SARS‐CoV‐2 S protein together with pHIV‐EGFP vector that encoded eGFP to track cell fusion events. After 24 h of plasmid transfection, the labeled 293T cells were combined with diluted serum in a ratio and cultured for a 2‐h duration under 37°C. The 293T‐ACE2 cells that expressed the ACE2 receptor were introduced into the mixture of 293T cells and sera. After coculturing for 2 h under 37°C, a microscope was utilized to observe cell fusion.

### 4.18 Statistical analysis

GraphPad Prism 9.0.0 was employed for statistical analysis. *p* Values are denoted within figures. One‐way ANOVA tests were used for comparing differences between each group. *p *< 0.05 was considered significant. Additional details are described in the Supporting Information.

## AUTHOR CONTRIBUTIONS

Wei Pang and Yong‐Tang Zheng developed the conceptual ideas and designed the study. Ying Lu, An‐Qi Li, Fan Shen, Wen‐Qiang He, Shu‐Heng Yu, Xiao‐Li Feng, and Ming‐Hua Li performed the experiments. Yan‐Bo Zhao, An‐Qi Li, and Songying Ouyang performed protein structure analysis. Ying Lu, An‐Qi Li, Fan Shen, and Wei Pang analyze the data. Ying Lu, An‐Qi Li, Fan Shen, Wei Pang, and Yong‐Tang Zheng drafted the original paper. Yong‐Tang Zheng and Wei Pang revised and edited this paper. All authors have read and approved the final manuscript.

## CONFLICT OF INTEREST STATEMENT

Ying Lu, Fan Shen, Yong‐Tang Zheng, and Wei Pang are listed as inventors on the Chinese patent related “A recombinant fusion protein derived from HR domains in S2 subunit of SARS‐CoV‐2 and its application (authorization number: ZL202111167024.2 and PCT/CN2022/135269)” to this work. The patents ZL202111167024.2 and PCT/CN2022/135269 have been filed that cover HR212 and its application presented here. Yong‐Tang Zheng and Wei Pang are the consultants in EterniVax Biomedical Inc., but have no potential relevant financial or nonfinancial interests to disclose. All other authors declare no financial or nonfinancial competing interests.

## ETHICS STATEMENT

The study was approved by the internal review board of Kunming Institute of Zoology, Chinese Academy of Sciences (approval number: SMKX‐2021‐01‐006). Human serum samples used in this study were obtained with informed consents. All experiments were carried out in accordance with relevant guidelines and regulations.

## Supporting information



Supporting Information

## Data Availability

The datasets used in the current study are available from the corresponding author on reasonable request.
