# Supplementary material for: Recombinant protein HR212 targeting heptad repeat 2 domain in spike protein S2 subunit elicits broad‐spectrum neutralizing antibodies against SARS‐CoV‐2 and its variants
Source: MedComm (2020). 2025 Feb 9;6(2):e70088. doi: 10.1002/mco2.70088 (PMC11808191; doi:10.1002/mco2.70088)
Supplement: Supplementary file 1 — Supporting Information [file MCO2-6-e70088-s001.docx]

Manuscript ID MCO2-2024-1393 R2

**Supplemental Information for:**

**Recombinant protein HR212 targeting heptad repeat 2 domain in spike protein S2 subunit elicits broad-spectrum neutralizing antibodies against SARS-CoV-2 and its variants**

**Authors:** Ying Lu, An-Qi Li, Fan Shen, Wen-Qiang He, Shu-Heng Yu, Yan-Bo Zhao, Xiao-Li Feng, Ming-Hua Li, Songying Ouyang, Yong-Tang Zheng, Wei Pang

**Table S1.** SARS-CoV-2-S circulating variants constructed in this study.

| SARS-CoV-2-S circulating variants | [Mutation](file:///F:\\Nature论文\\Nature\\22-5-2\\Supplementary_Materials.docx#/javascript:;) [site](file:///F:\\Nature论文\\Nature\\22-5-2\\Supplementary_Materials.docx#/javascript:;)s |
| --- | --- |
| B.1.617.1 | G142D, E154K, L452R, E484Q, D614G, P618R, Q1071H, and H1101D |
| B.1.617.2.V2 | T19R, G142D, E156G, 157-158 FR deletion (ΔFR157-158), L452R, T478K, D614G, P681R and D950N |
| B.1.429 | S13I, W152C, and L452R |
| B.1.525 | Q52R, A67V, ΔHV69-70, ΔY145, E484K, D614G, Q677H, and F888L |
| B.1.526 | L5F, T95I, D253G, E484K, D614G, and A701V |
| B.1.617 | E154K, L452R, E484Q, and P681R |
| B.1.1.7 (Alpha) | Δ69–70 HV, Δ144 Y, N501Y, A570D, D614G, P681H, T761I, S982A, and D1118H |
| B.1.351 (Beta) | L18F, D80A, D215G, ΔLAL242-244, K417N, E484K, N501Y, D614G, and A701V |
| B.1.1.28 (Gamma) | L18F, T20N, P26S, D138Y, R190S, K417T, E484K, N501Y, D614G, and H655Y. |
| B.1.617.2 (Delta) | T19R, G142D, L452R, T478K, D614G, P681R and D950N |
| C.37 (Lambda) | G75V，T76I，ΔR246，ΔS247，ΔY248，ΔL249，ΔT250，ΔP251，ΔG252，D253N，L452Q，F490S，D614G，and T859N |
| B.1.621 (Mu) | T95I, Y146insN, R346K, E484K, N501Y, D614G, P681H, and D950N |
| B.1.1.529 (Omicron BA.1) | A67V, ΔHV69-70, T95I, ΔGVY142-144, Y145D, ΔN211, L212I, ins214EPE, G339D, S371L, S373P, S375F, K417N, N440K, G446S, S477N, T478K, E484A, Q493R, G496S, Q498R, N501Y, Y505H, T547K, D614G, H655Y, N679K, P681H, N764K, D796Y, N856K, Q954H, N969K, and L981F |
| Omicron BA.2 | T19I; ΔL24, ΔP25, ΔP26, A27S, G142D, V213G, G339D, S371F, S373P, S375F, T376A, D405N, R408S, K417N, N440K, S477N, T478K, E484A, Q493R, Q498R, N501Y, Y505H, D614G, H655Y, N679K, P681H, N764K, D796Y, Q954H, and N969K |
| Omicron BA.3 | A67V, Δ69-70, T95I, G142D, ΔV143, ΔY144, ΔY145, ΔN211, L212I, G339D, S371F, S373P, S375F, D405N, K417N, N440K, G446S, S477N, T478K, E484A, Q493R, Q498R, N501Y, Y505H, D614G, H655Y, N679K, P681H, N764K, D796Y, Q954H, and N969K |
| Omicron BA.4/5 | T19I, L24S, Δ25-27, Δ69-70, G142D, V213G, G339D, S371F, S373P, S375F, T376A, D405N, R408S, K417N, N440K, L452R, S477N, T478K, E484A, F486V, Q498R, N501Y, Y505H, D614G, H655Y, N679K, P681H, N764K, D796Y, Q954H, and N969K |
| SARS-CoV-2-S circulating variants | [Mutation](file:///F:\\Nature论文\\Nature\\22-5-2\\Supplementary_Materials.docx#/javascript:;) [site](file:///F:\\Nature论文\\Nature\\22-5-2\\Supplementary_Materials.docx#/javascript:;)s |
| Omicron BQ.1 | T19I, L24S, Δ25-27, Δ69-70, G142D, V213G, G339D, S371F, S373P, S375F, T376A, D405N, R408S, K417N, K439T, N440K, N455K, L452R, S477N, T478K, E484A, F486V, Q498R, N501Y, Y505H, D614G, H655Y, N679K, P681H, N764K, D796Y, Q954H, and N969K |
| Omicron XBB.2.3 | T19I, ΔL24, ΔP25, ΔP26, A27S, V83A, G142D, ΔY144, H146Q, E180V, Q183E, V213E, G252V, G339H, R346T, L368I, S371F, S373P, S375F, T376A, D405N, R408S, K417N, N440K, V445P, G446S, N460K, S477N, T478R, E484A, F486P, F490S, Q498R, N501Y, Y505H, P521S, T547I, D614G, H655Y, N679K, P681H, N764K, D796Y, Q954H, and N969K |
| Omicron EG.5 | T19I, ΔL24, ΔP25, ΔP26, A27S, V83A, G142D, ΔY144, H146Q, Q183E, V213E, G252V, G339H, R346T, L368I, S371F, S373P, S375F, T376A, D405N, R408S, K417N, N440K, V445P, G446S, F456L, N460K, S477N, T478K, E484A, F486P, F490S, Q498R, N501Y, Y505H, Q613H, D614G, H655Y, N679K, P681H, N764K, D796Y, Q954H, and N969K |
| Omicron BA.2.86 | T19I, R21T, L24S, Δ25/27, S50L, Δ69/70, V127F, G142D, Δ144/144, F157S, R158G, N211I, Δ212/212, V213G, L216F, H245N, A264D, I332V, G339H, K356T, S371F, S373P, S375F, T376A, R403K, D405N, R408S, K417N, N440K, V445H, G446S, N450D, L452W, N460K, S477N, T478K, N481K, E484K, F486P, Q498R, N501Y, Y505H, E554K, A570V, D614G, P621S, H655Y, N679K, P681R, N764K, D796Y, S939F, Q954H, N969K, P1143L |
| Omicron JN.1 | T19I, R21T, L24S, Δ25/27, S50L, Δ69/70, V127F, G142D, Δ144/144, F157S, R158G, N211I, Δ212/212, V213G, L216F,H245N, A264D, I332V, G339H, K356T, S371F, S373P, S375F, T376A, R403K, D405N, R408S, K417N, N440K, V445H, G446S, N450D, L452W, L455S, N460K, S477N, T478K, N481K, Δ483/483, E484K, F486P, Q498R, N501Y, Y505H, E554K, A570V, D614G, P621S, H655Y, N679K, P681R, N764K, D796Y, S939F, Q954H, N969K, P1143L |

**Table S2.** Fold changes of rabbit anti-HR212 serum neutralization titers against different variants compare to that against Wuhan-Hu-1 strain.

| SARS-CoV-2 variants pseudovirus | Fold change (variants/Wuhan-Hu-1) |
| --- | --- |
| S477N | 0.62 |
| E484K | 0.76 |
| A222V | 1.21 |
| N439K | 0.75 |
| K417N | 7.41 |
| D839Y | 0.42 |
| D614G | 1.64 |
| B.1.617.1 | 7.79 |
| B.1.617.2.V2 | 0.67 |
| B.1.429 | 3.05 |
| B.1.525 | 0.9 |
| B.1.526 | 0.67 |
| B.1.617 | 2.59 |
| B.1.1.7 (Alpha) | 1.5 |
| B.1.351 (Beta) | 13.13 |
| B.1.1.28 (Gamma) | 0.45 |
| B.1.617.2 (Delta) | 0.5 |
| C.37 (Lambda) | 2.34 |
| B.1.621 (Mu) | 0.36 |
| B.1.1.529 (Omicron BA.1) | 0.36 |
| Omicron BA.2 | 0.32 |
| Omicron BA.3 | 1.13 |
| Omicron BA.4/5 | 0.78 |
| Omicron BQ.1 | 0.12 |
| Omicron XBB 2.3 | 0.14 |
| Omicron EG.5 | 0.07 |
| Omicron BA.2.86 | 0.15 |
| Omicron JN.1 | 0.37 |

**
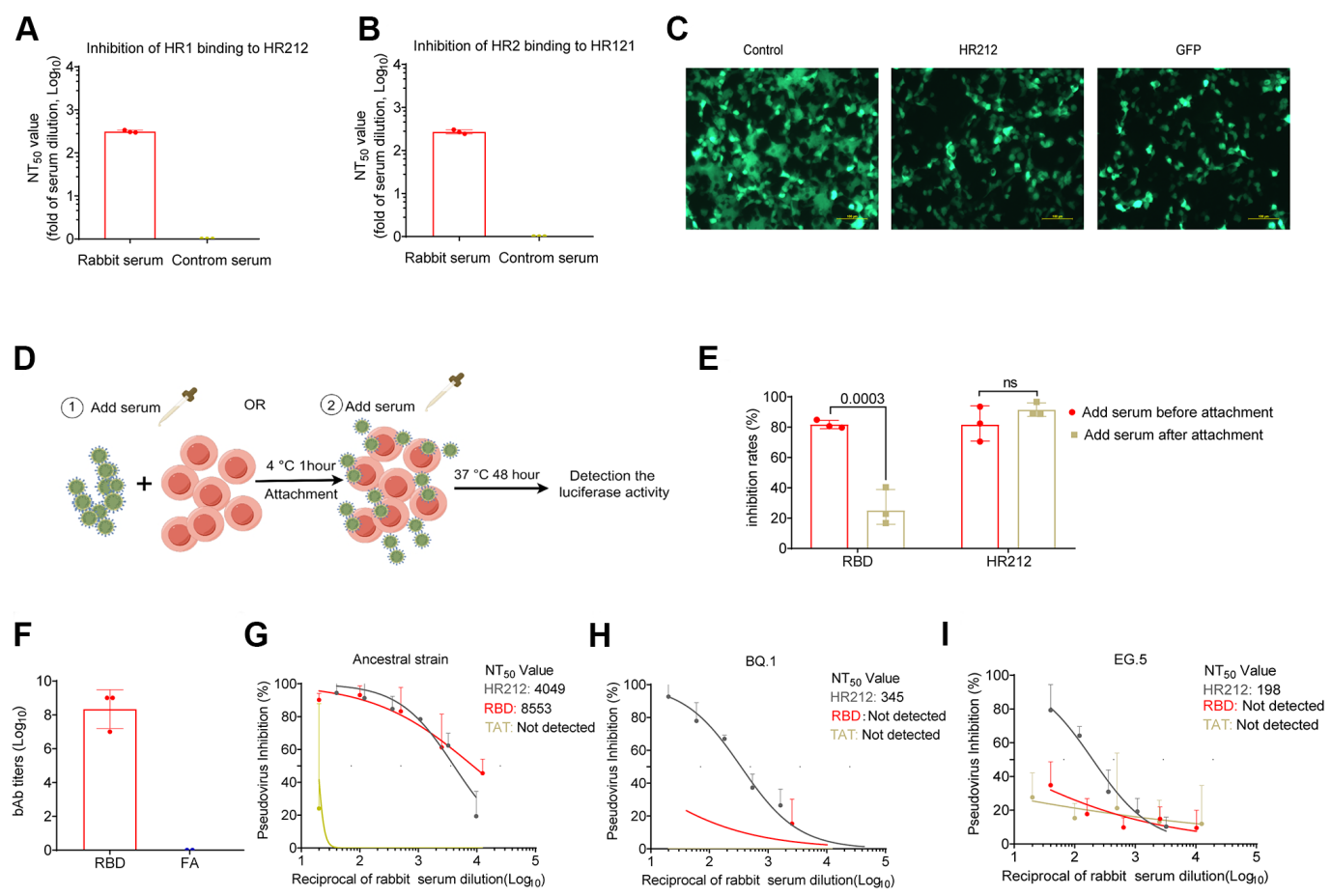
Figure S1.** The antibody titers and neutralizations of rabbit sera against SARS-CoV-2 *in vitro*. (A) Rabbit anti-HR212 sera (n = 3) could inhibit the binding between HR212 and HR1 when sera were added to the coated HR121, followed washing and then added HR1-HRP. (B) Rabbit anti-HR212 sera (n = 3) could inhibit the binding between HR121 and HR2 when serum-HR2-HRP mixture were added to the coated HR121. (C) Fluorescent photograph of spike of SARS-CoV-2 ancestral strain mediated cell-cell fusion in the presence of HR212 serum. Representative fluorescent figures were selected randomly. (D-E). Anti-HR212 serum could still block the entry of the virus even after the virus adsorption. (F) End-point titers of RBD- bAbs in RBD immunized rabbits (n = 3). (G-I) Inhibitory effect of rabbit serum anti-HR212, anti-RBD and anti-HIV-1 Tat (a protein from HIV-1 served as an unrelated protein control) against ancestral strain, BQ.1 and EG. 5 variants. In (A), (B), and (E-I), data are presented as geometric mean ± geometric SD and differences between each group are determined by one-way ANOVA. RBD: Receptor binding domain; FA: Freund's adjuvant.


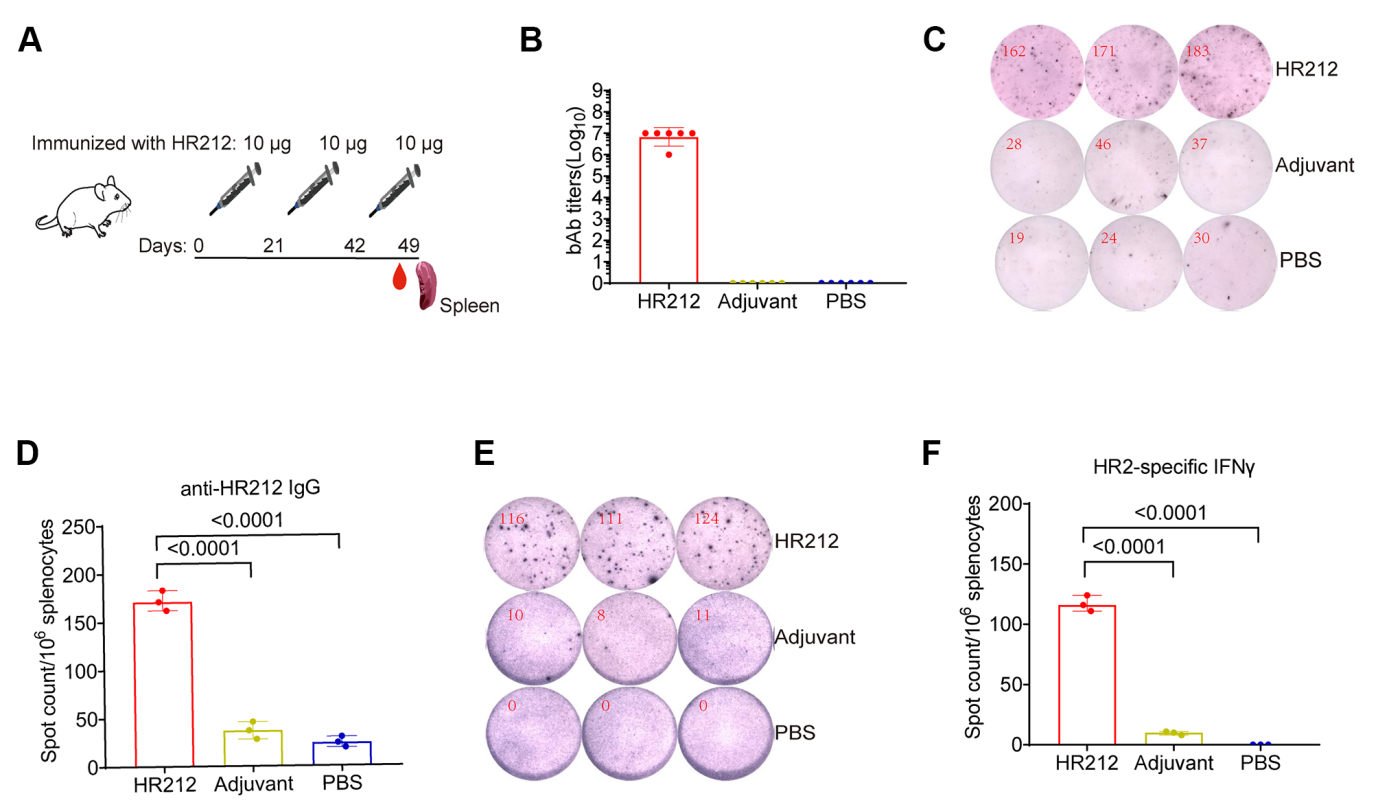


**Figure S2.** HR212 induced humoral and cell-mediated immune responses in BALB/c mice. (A) Schematic diagram of BALB/c mice immunized with HR212 at three-week intervals. In each group, n = 6. (B) Titers of bAbs at 7 days after the third immunization. At 7 days after the third immunization in each group, HR212-specific humoral immunity (C and D) and HR2-specific cytotoxic T reaction (E and F) were evaluated using ELISPOT Kits, n = 3. In (B), data are presented as geometric mean ± geometric SD. In (D) and (F), data are presented as median ± interquartile range, and differences between each group are determined by one-way ANOVA.


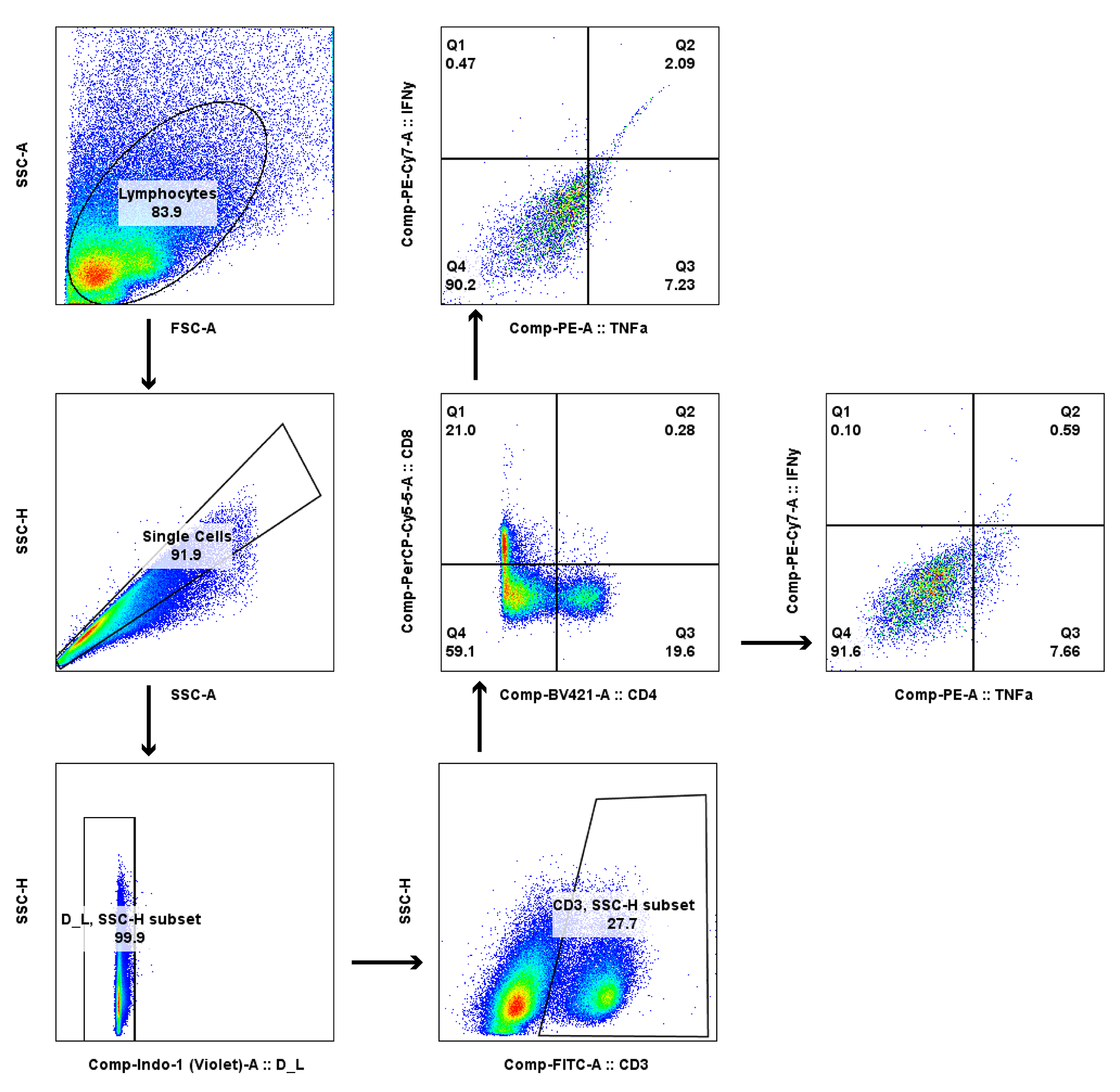


**Figure S3.** Gating strategy for T cells in the spleens. Depicted figures displayed the gating path of a spleen sample from a mouse immunized with HR212 plus Freund's adjuvant.


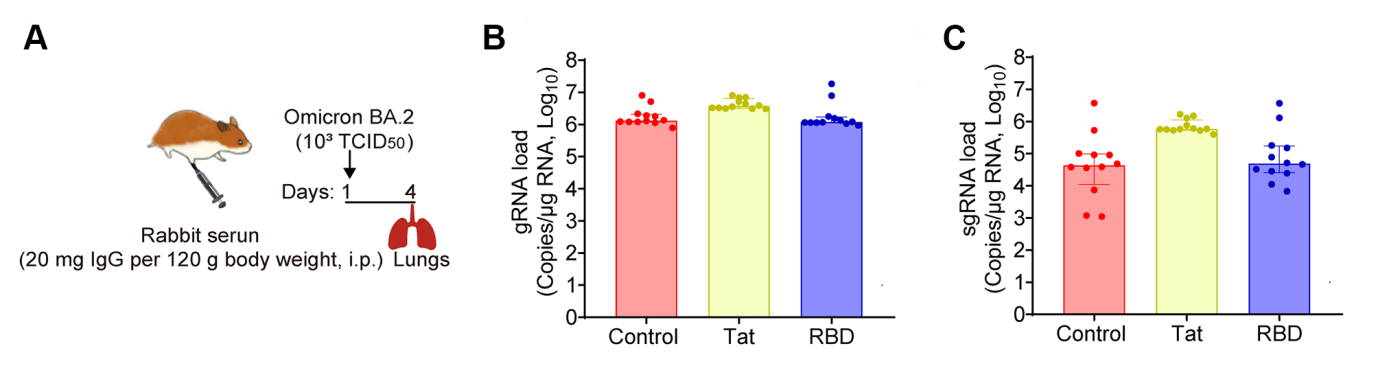


**Figure S4.** Passive immunization with rabbit anti-RBD or anti-HIV-1 Tat sera did not protect Syrian golden hamsters from Omicron BA.2 variant infection. (A). Omicron BA.2 (TCID_50_ = 10^3^) challenge was carried out 24 h after passive immunization with the anti-sera (n= 12). (B) Quantitative PCR was used to analyze Omicron BA.2 genomic RNAs (gRNAs). and (C) subgenomic RNAs (sgRNAs). In (B) and (C), data are presented as median ± interquartile range. TCID_50_：Tissue culture infective dose.
